# Human Bone Marrow-Derived Mesenchymal Stromal Cell-Seeded Bone Biomaterial Directs Fast and Superior Mandibular Bone Augmentation in Rats

**DOI:** 10.1038/s41598-019-48236-8

**Published:** 2019-08-14

**Authors:** Daniel Deluiz, Gaëtan J.-R. Delcroix, Gianluca D’Ippolito, Cristina Grau-Monge, Andrea Bonnin-Marquez, Teresita Reiner, Eduardo M. B. Tinoco, Thaís Amadeu, Fabio R. Pires, Paul C. Schiller

**Affiliations:** 1 Department of Periodontology, State University of Rio de Janeiro, Rio de Janeiro, RJ Brazil; 2 Geriatric Research, Education, and Clinical Center, and Research Service, Bruce W. Carter Department of Veterans Affairs Medical Center, Miami, FL USA; 3 Nova Southeastern University, College of Allopathic Medicine, Fort Lauderdale, FL USA; 4 Department of Orthopaedics, University of Miami Miller School of Medicine, Miami, FL USA; 5 Department of Biomedical Engineering, College of Engineering, University of Miami, Miami, FL USA; 6 Department of Pathology and Laboratories, State University of Rio de Janeiro, Rio de Janeiro, RJ Brazil; 7 Department of Oral Pathology, State University of Rio de Janeiro, Rio de Janeiro, RJ Brazil; 8 Department of Biochemistry & Molecular Biology and Medicine, University of Miami Miller School of Medicine, Miami, FL USA

**Keywords:** Mesenchymal stem cells, Dental biomaterials, Stem-cell research

## Abstract

Atrophic maxillary ridges present a challenge in the field of oral implantology. Autologous bone is still considered the gold standard grafting material, but the increased morbidity and surgical complications represent a major drawback for its use. The aim of this study was to assess the efficacy of an off-the-shelf cell-seeded bone biomaterial for mandibular bone augmentation, compared to its acellular counterpart. We used a rat model to test the osteogenic properties of bone marrow-derived mesenchymal stromal cells (MSCs)-seeded bone microparticles compared to acellular bone microparticles alone. Rats were euthanized at 4 and 8 weeks, and results analyzed using micro-CT imaging, histology (H&E, Masson’s Trichrome), histomorphometry and immunohistology (Tartrate-Resistant Acid Phosphatase-TRAP, Osteocalcin and human specific anti-mitochondria antibodies). Micro-CT analysis demonstrated that the cell-seeded biomaterial achieved significantly more bone volume formation at 4 weeks (22.75 ± 2.25 mm^3^ vs 12.34 ± 2.91 mm^3^, p = 0.016) and at 8 weeks (64.95 ± 5.41 mm^3^ vs 42.73 ± 10.58 mm^3^, p = 0.029), compared to the acellular bone microparticles. Histology confirmed that the cell-seeded biomaterial was almost completely substituted at 8 weeks, in opposition to the acellular biomaterial group. Immunohistochemical analysis showed a significantly higher number of TRAP and Osteocalcin positive cells at 4 weeks in the cell-seeded group compared to the acellular group, thereby demonstrating a higher rate of bone remodeling in the presence of MSCs. The grafted human cells remained viable and were detected up to at least 8 weeks, as observed using the human specific anti-mitochondria antibody. This off-the-shelf material available in unlimited quantities could therefore represent a significant advance in the field of mandibular bone augmentation by providing a larger volume of new bone formation in a shorter time.

## Introduction

Bone autografts usually demonstrate high success rates in alveolar reconstructions and are thus the current gold standard grafting material^1–3^. There is however an increasing need for other grafting materials due to the limited availability, the increased morbidity and complications associated with autograft harvesting^4–8^.

In the last decade, studies reported the use of cadaveric bone allografts to replace autografts^9–15^. Compared to autografts, the allogeneic bone shows slower incorporation and higher volumetric resorption^16,17^. Furthermore, studies suggest a poorer engraftment into the host tissue of allogeneic-based oral grafts^18^. There are also controversial results as several reports showed living and newly formed bone incorporated in the grafted areas^10,13,19–21^, while others have demonstrated inadequate revascularization, little substitution, and a small number of cells remaining in the remodeling process^17,22^.

The attempts of improving bone grafts using MSCs have shown good preliminary outcomes evidenced by clear differences between the enriched grafts and the non-cellularized grafts^23–25^. Our group developed a cellularized bone substitute material which is currently available on the US market for spine fusion applications (ViaGraft^®^, Vivex Biomedical, Miami, FL)^26^. This material is made of cadaveric human bone microparticles (30% cortical bone, 30% cancellous bone and 40% fully demineralized cortical bone) ranging from 100–300 microns in size and seeded with MSCs (see supplementary Fig. S1 for CD marker expression analyzed by flow cytometry). This product contains on average 220,000 cells/cc of material.

The hypothesis of this study is that an off-the-shelf cell-seeded bone biomaterial will achieve a better incorporation in the context of jaw’s bone augmentation compared to the same, but acellular, biomaterial.

## Results

### Surgical procedure

**Figure 1:**
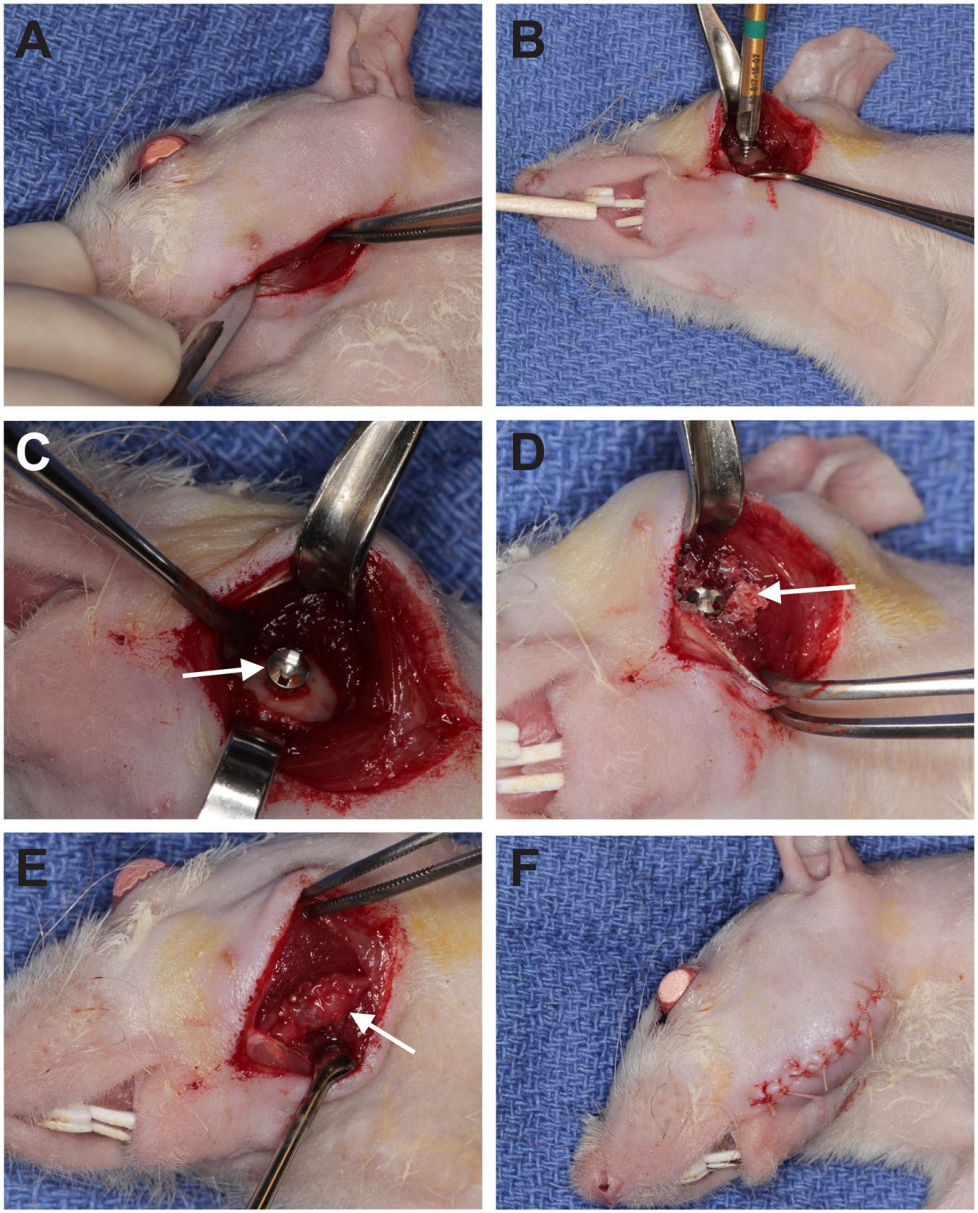
Rat model of alveolar bone augmentation: surgical procedure. Submandibular incision exposing the masseter muscle and dissection of the muscle until reaching the lateral aspect of the mandible (**A**); placement of the titanium screw (**B**); fixed titanium screw and exposed host bone ready to receive the graft (**C**); bone graft stable on the reception site (**D**); amniotic membrane covering the entire grafted area (**E**); surgical wound fully closed by sutures (**F**).

The surgical procedure used is described in Fig. 1 and in greater details in the material and methods section.

### Cellular content increased the volume of new bone formation and promoted faster bone microparticle remodeling

**Figure 2:**
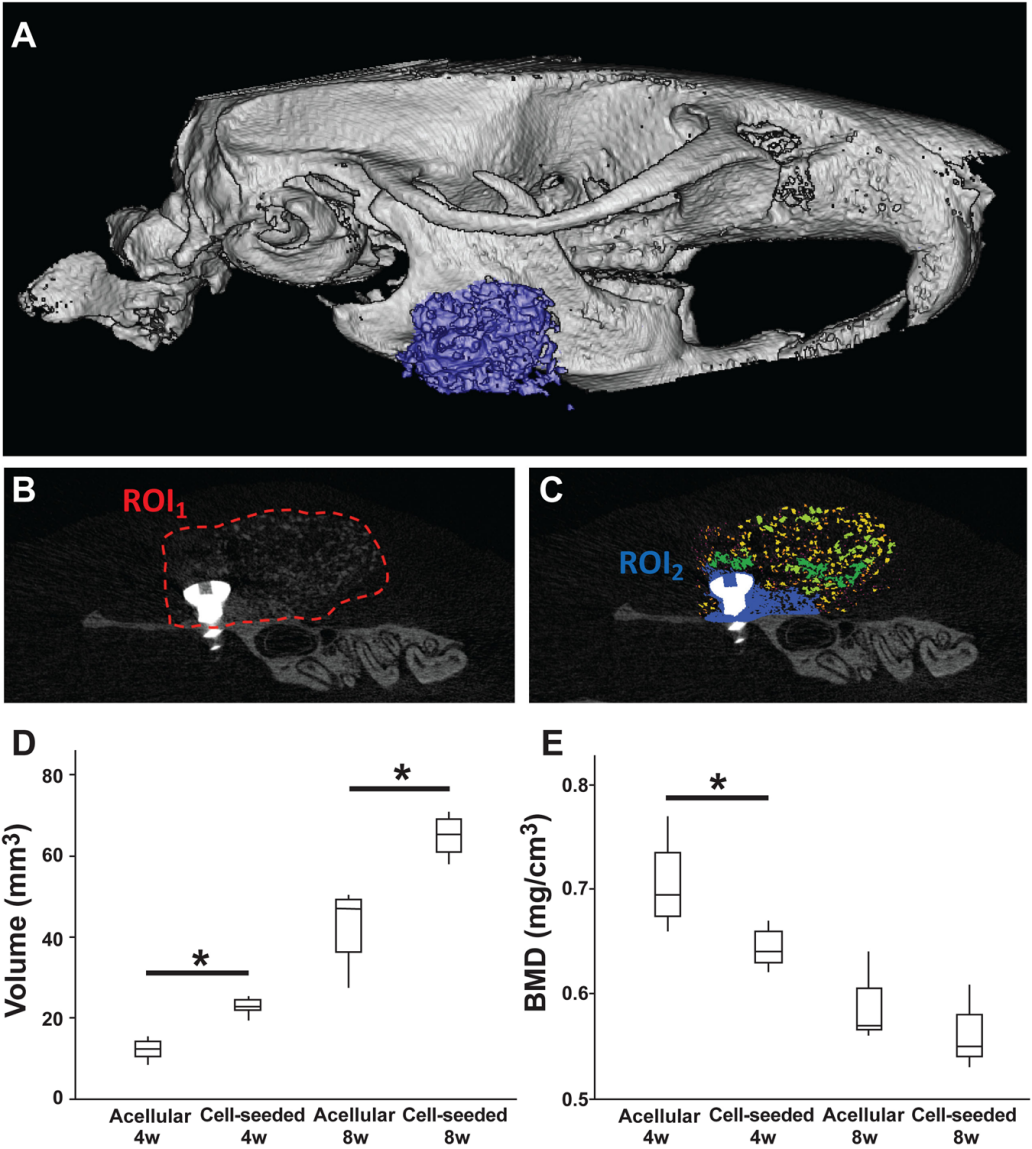
Micro-CT analysis demonstrated a higher volume of new bone formation and a lower bone mineral density in the cell-seeded biomaterial treated group. 3D reconstruction of the grafted area (**A**). The screw and the grafted bone are clearly visible on (**B**) where the region of interests (ROI_1_) is delineated in red. In blue (**C**) is the incorporated new bone formation (ROI_2_) and in yellow/green are the unincorporated excess bone microparticles. ROI_2_ was analyzed to generate the graphs (**D**,**E**). The cell-seeded biomaterial caused a higher volume of new bone formation (**D**) compared to the acellular biomaterial, both at 4 and 8 weeks post-surgery (p = 0.016 and 0.029, respectively). Bone mineral density (BMD) in the entire grafted area decreased with time due to grafted bone remodeling (**E**). Bone mineral density was lower in the case of the cell-seeded biomaterial at 4 weeks post-surgery, probably due to a higher rate of remodeling (p = 0.032). At 8 weeks, no difference could be found (p = 0.20).

Strikingly, the cell-seeded biomaterial group achieved significantly (p = 0.016) more bone volume gain compared to the acellular bone microparticles group. The cell-seeded biomaterial treated group gained on average 22.75 ± 2.25 mm^3^ of bone, while the average volume gain was only 12.34 ± 2.91 mm^3^ in the acellular microparticles group at 4 weeks. At 8 weeks, the volume of cell-seeded biomaterial bone formation increased to 64.95 ± 5.41 mm^3^ while the acellular biomaterial group reached only 42.73 ± 10.58 mm^3^, which was also statistically different (p = 0.029). The average bone mineral density (BMD) of the grafted material decreased with time in both groups, as it is expected to happen during a normal bone remodeling process. However, even though there was no significant difference in the BMD at 8 weeks between the 2 groups (the cell-seeded biomaterial group was 0.56 ± 0.03 mg/cm^3^ versus 0.59 ± 0.04 mg/cm^3^ for the acellular group, Fig. 2), the decrease in bone mineral density happened faster in the cell-seeded biomaterial group compared to the acellular group. Indeed, the BMD at 4 weeks was 0.64 ± 0.02 mg/cm^3^ for the cell-seeded biomaterial treated group, versus 0.71 ± 0.05 mg/cm^3^ for the acellular biomaterial group (p = 0.032).

### Grafted MSCs improved the maturity and stability of the newly-formed bone

**Figure 3:**
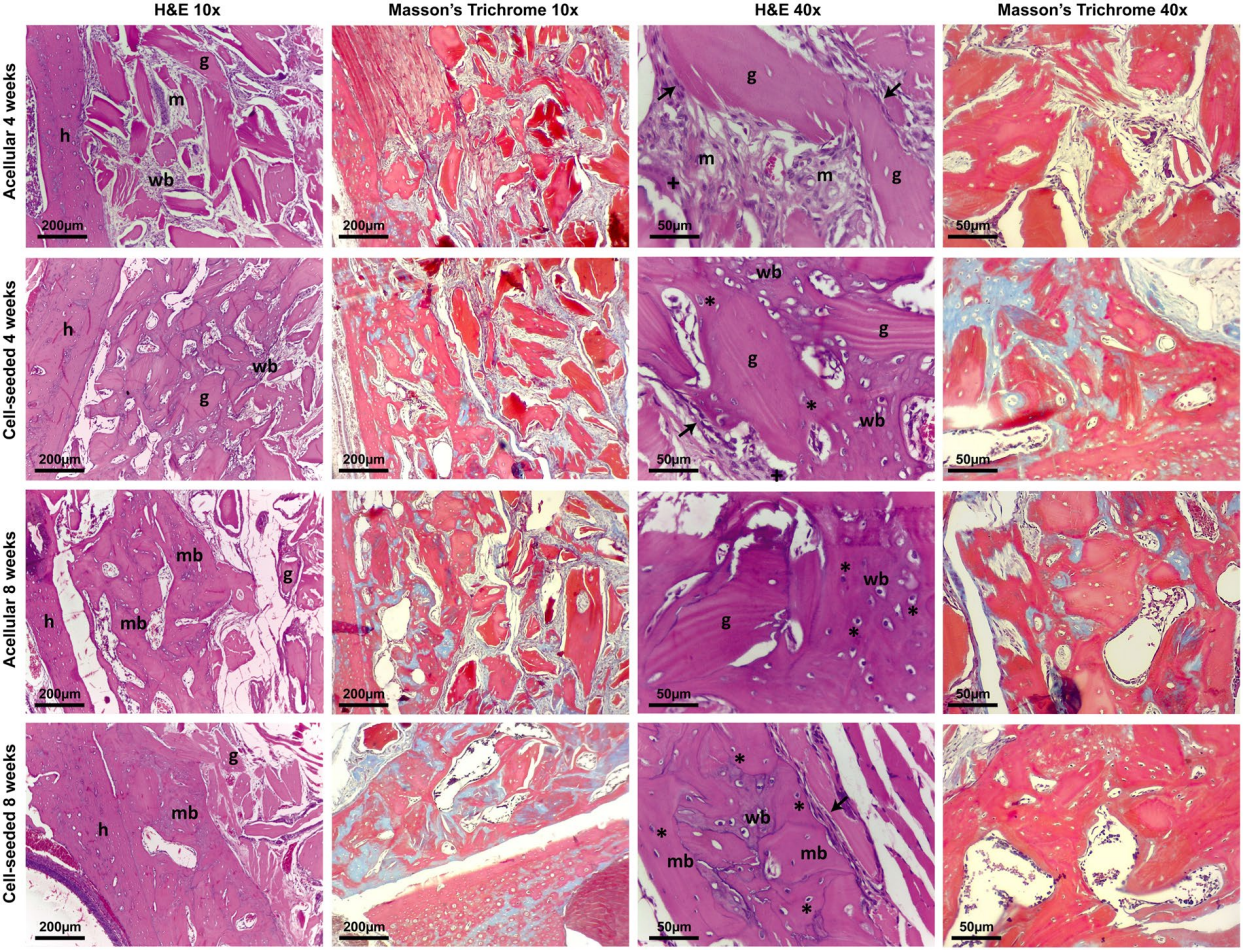
Histologic analysis demonstrated higher bone remodeling and new bone formation in the cell-seeded biomaterial treated group. At 4 weeks, bone matrix deposition and fibroblast-like cells are observed around the bone microparticles of the cell-seeded biomaterial group, while the graft particles remain largely unincorporated in the acellular biomaterial group. In the acellular biomaterial group at 8 weeks, woven bone deposition is observed around the graft particles, which are not yet completely substituted. In opposition, mature bone together with newly formed woven bone is observed in the cell-seeded biomaterial treated group at 8 weeks, in which the microparticles were almost completely substituted. Most importantly, the incorporated material and the host bone consisted of a solid block unit in the cell-seeded biomaterial treated group at 8 weeks. The 2 left columns are low magnification (10x) and the 2 right columns are high magnification (40x). (Legend: h: host bone; nw: new woven bone; m: bone matrix; mb: new mature bone; g: graft particle, arrows: osteoblasts, “*”: osteocytes, “+”: osteoclasts).

Histological analyses (H&E and Masson’s Trichrome staining, Fig. 3) confirmed the higher rate of bone remodeling and the larger fraction of new bone within the graft area when the bone microparticles are combined with MSCs. Osteoclasts were observed close to the interface between the host bone and the graft as well as around the graft microparticles (Fig. 3, crosses). Osteoblasts were evident lining the edges of the microparticles (Fig. 3, arrows). At 4 weeks, the control acellular microparticle group exhibited bone matrix deposition with fibroblast-like cells around the material microparticles (Fig. 3) while in the cell-seeded biomaterial treated group it was possible to see new woven bone deposition around the grafted material creating “bone bridges” connecting the microparticles (Fig. 3). This was only observable at 8 weeks around some of the microparticles in the acellular biomaterial group (Fig. 3) in which the unincorporated granules were still clearly recognizable and presented empty osteocytes lacunae. At 8 weeks, new mature bone together with newly formed woven bone was observed in the cell-seeded biomaterial treated group (Fig. 3). Osteocytes were observed entrapped within the calcified tissue predominantly in the cell-seeded group (Fig. 3, stars). In the incorporated segment of the cell-seeded group, the graft was almost completely substituted with living bone and unincorporated microparticles were scarcely present. Most importantly, the incorporated material and the host bone presented as a single, solid bone block unit (Fig. 3), a critical point for implant placement in humans. Noteworthy, no inflammatory cells were observed in any groups, as observed on the H&E sections.

**Figure 4:**
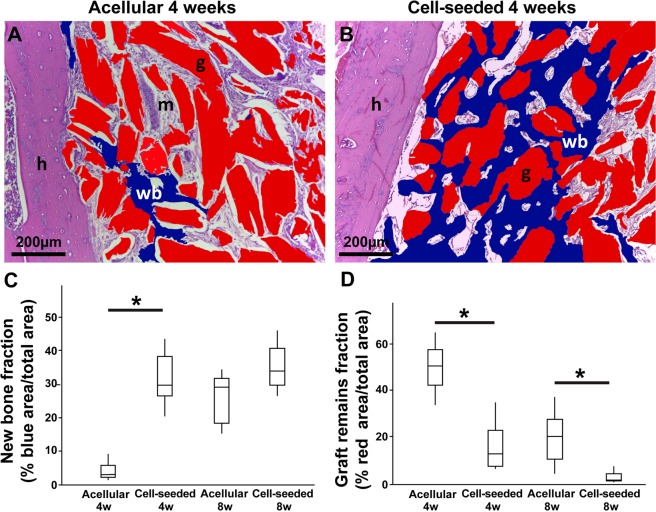
The faster rate of bone remodeling was confirmed by histomorphometric analysis. Panels A and B are representative images (taken from Fig. 3A,C) depicting how the analysis was performed. In red are the remaining graft microparticles, in blue the newly formed bone. ROI and analysis were performed using the ImageJ software. The fraction of new bone was significantly higher in the cell-seeded biomaterial group at 4 weeks, indicating a faster osteogenesis (p = 0.012). The remaining graft particles were significantly lower in the cell-seeded biomaterial treated group at both time points indicating a higher substitution/resorption (p = 0.024) (**D**). At 8 weeks, the fraction of new bone within the graft was not significantly different between the 2 groups (**C**). Legend: h: host bone; wb: new woven bone; m: bone matrix; g: graft particle.

To complement the micro-CT scan analysis that was used to provide information on the total bone volume gain and BMD (Fig. 2), we also performed histomorphometric analysis using ImageJ software to assess the quality of the new bone being formed, by calculating the new bone fraction and graft remains fraction. The fraction of newly formed bone within the graft area in the cell-seeded biomaterial treated group at 4 weeks was significantly higher than in the acellular biomaterial group (31.45 ± 7.88% vs 4.41 ± 3.76%, respectively [p = 0.012]), in blue in Fig. 4A,B. No difference was found at 8 weeks in the fraction of new bone present in the graft (35.06 ± 8.15% vs 25.50 ± 8.24%, for the cell-seeded and acellular groups, respectively), while we demonstrated using the CT-scan (Fig. 2D), that the overall volume of new bone formation was higher at both 4 and 8 weeks for the cell-seeded group. The cell-seeded biomaterial treated group also presented significantly less remaining graft particles at both time points (16.03 ± 10.18% vs 49.52 ± 15.53%, at 4 weeks and 2.88 ± 5.76% vs 19.75 ± 11.91% at 8 weeks [p = 0.024]), in red in Fig. 4A,B.

### Cellular content increased the frequency of TRAP and Osteocalcin positive cells in the newly-formed bone

**Figure 5:**
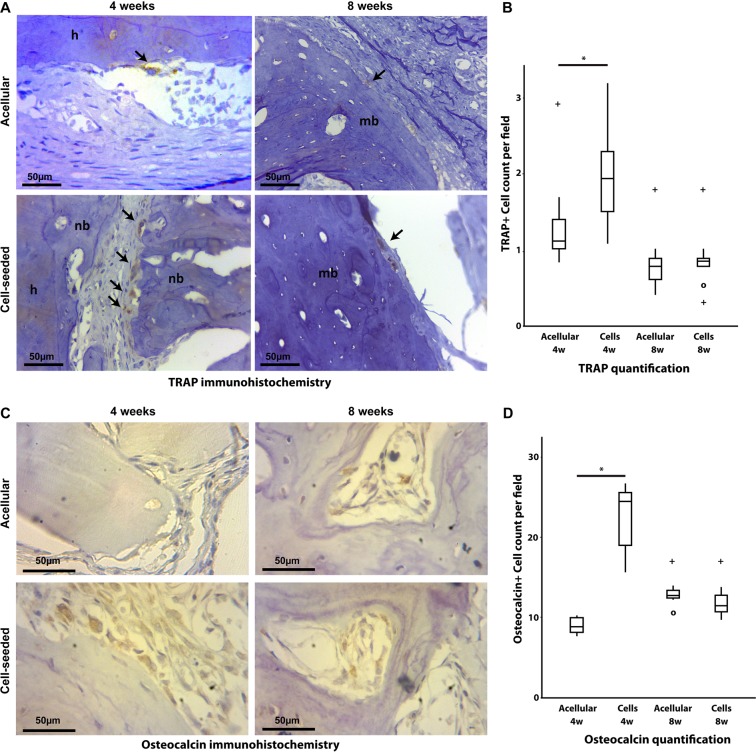
Cellular content increased the frequency of TRAP and Osteocalcin positive cells in the newly-formed bone. The cell-seeded biomaterial treated group showed significantly more TRAP and Osteocalcin positive cells at 4 weeks compared to the acellular biomaterial (p = 0.015 for TRAP and p < 0.01 for Osteocalcin) thereby demonstrating higher remodeling activity at the earlier time point. No significant differences between TRAP and Osteocalcin were detected at 8 weeks. “+” indicates an outlier and “o” indicates more than one outlier.

TRAP and Osteocalcin immunohistochemical analysis were performed to quantify the number of osteoclasts and osteoblasts present in the newly formed tissue, respectively. TRAP and Osteocalcin were detected in all groups at both time points. However, the cell-seeded biomaterial treated group showed significantly more TRAP and Osteocalcin positive cells count at 4 weeks (Fig. 5) compared to the acellular biomaterial group (p = 0.015 for TRAP and p < 0.01 for Osteocalcin), thereby demonstrating higher remodeling activity at the earlier time point. There was no significant difference between both groups at 8 weeks for both markers (Fig. 5B,D).

### Human cells are detected *in vivo* up to at least 8 weeks after grafting

**Figure 6:**
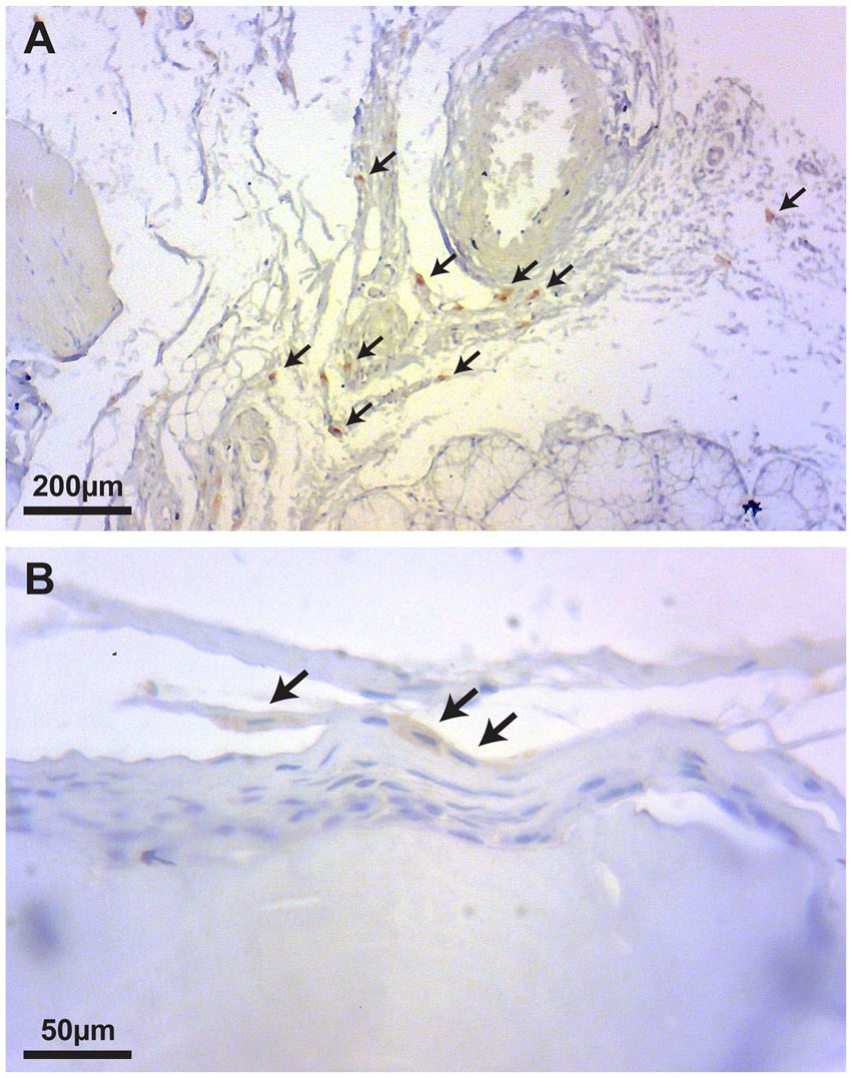
Human cells are detected up to at least 8 weeks after grafting. Human-specific anti-mitochondria antibody was used to detect the presence of human cells within the host tissue. Many human cells are detected in the tissue at 4 weeks (**A**), and cells of human origin appeared to be perfectly integrated in the tissue at 8 weeks (**B**).

Cells presenting positive immunolabeling for human mitochondria were found at both 4 and 8 weeks after grafting in the cell-seeded treated group (Fig. 6). We also confirmed that the human mitochondria staining appeared fully negative in the acellular group (data not shown).

## Discussion

Bone regeneration of the jaws prior to dental implants placement is a major challenge in modern dentistry^2,27^. The aim of alveolar ridge reconstruction is to provide sufficient structure for fixation of the implants as well as a healthy and physiologically active environment for osseointegration. To achieve this goal, the grafting material should be capable of replacing the lost tissue with a morphology and mechanical properties as similar as possible to the native bone^2,27^. In this study, we showed that a cell-seeded bone biomaterial is capable of consistently and successfully augmenting the mandibular bone, while presenting histological features of healthy living bone. Bone biomaterials have been safely used in the field of Orthopaedics surgery in the past 40 years^28^, and the new generation of viable bone biomaterial we tested in this study is currently being used for applications such as spine fusion with great success^26^. In addition, the remodeling of this cell-seeded bone biomaterial was more rapid and led to a larger bone formation compared to the acellular biomaterial used as a control. Importantly, we noted almost no remaining graft particles with the cell-seeded biomaterial at 8 weeks, which is something usually seen only with autografts^29–31^.

The present study uses a mandibular horizontal augmentation model to assess the osteogenic (bone-forming) properties of the cell-seeded biomaterial. This model is well-characterized and presents a similar situation to that found in the alveolar ridge atrophy where the new bone needs to be generated extra-skeletally by the placement of the graft on top of an essentially uninjured bone surface^32^, and is different from the “critical size defect” model in which the calvaria is mostly used. We compared the test group (cell-seeded biomaterial) to a corresponding biomaterial with no viable cells, thus assessing specifically the osteogenic potential contributed by the addition of the graft’s cellularized component. The addition of MSCs to the bone microparticles resulted in a significantly faster new bone formation demonstrating the beneficial bone forming properties of the cellularized biomaterial.

Bone substitutes incorporate via creeping substitution^33^. The engraftment and remodeling starts with angiogenesis, followed by the replacement of the biomaterial by the host bone. Thus, during the incorporation phase, the grafted area becomes a hybrid structure comprising the calcified original graft and the newly formed bone, variably mineralized^34^. In this study, we showed that the cell-seeded biomaterial can achieve effectively more bone volume formation compared to acellular bone microparticles. With the aid of the micro-CT scan, it was possible to have an accurate characterization of the portion of the graft that was integrated to the host bed (blue ROI in our study)^35^. Bone mineral density (BMD) of the grafts decreased during the time-course of the study. The cell-seeded biomaterial treated group BMD decreased faster than the control group, as observed at 4 weeks, while both groups reached a non-significantly different low level of BMD at 8 weeks, similar to that of native mandibular bone^35,36^. Similar changes in the BMD of several bone substitutes over the engraftment process are reported in the literature^35,37–41^. Engraftment comprises a sequence of bone remodeling cellular events that produce changes in the amounts of bone mineral content as well as the external calcium balance. The first phase of the remodeling process is the bone (or biomaterial) resorption and osteoid formation^42^. The result of the new bone production in this condition is the reduction of the relative mineralization due to the low mineralized nature of the osteoid^42,43^. The coupled process of bone removal and addition can result in small imbalances depending on the phase and on the degree of bone remodeling. Active remodeling sites lack some of their mineral content while the full mineralization requires time to be established^42^. Our findings show a time-dependent decrease of the BMD. Considering our histologic and histomorphometric data, we hypothesize that the inorganic phase of the grafted product decreased with time by resorption as substitution by the host bone proceeded, hence the lower BMD. It is known that high turnover states and increased osteoblast activity is associated with lower mineralization of bone as a result of the calcified tissue resorption before the secondary mineralization. In humans, the use of bone forming stimulating drugs, such as teriparatide, has shown a transient reduction of the mineralization due to the increase in bone turnover^44,45^. The existence of the transitory low-mineral bone components means that mineral-based measurements underestimate, to some degree, the amount and the nature of newly formed bony tissue^42^.

Tartrate-resistant acid phosphatase (TRAP) is a glycosylated monomeric metalloproteinase expressed in mammals and is one of the main markers for osteoclast activity^46^. Osteoclasts are usually evident on remodeling surfaces of new osteoid matrices and on bone grafts undergoing substitution^46,47^. Although osteoclastic activity is part of the physiological bone remodeling maintaining integrity of the adult skeleton, TRAP activity has been reported to be completely absent on native maxillary cortexes, indicating a very low rate of bone turnover in these sites^48^. Osteoclast cell lineage certainly plays the most significant role in the resorption and remodeling of bone substitute materials^49^, even though other cells are reported to also degrade products of biomaterials^46,50^. We showed here that the cell-seeded biomaterial presented significantly more TRAP-positive cells at 4-weeks compared to its acellular counterpart, thus promoting a more rapid and effective substitution in the early stages, and likely leading to a more mature-like higher-quality bone in a considerably shorter period. This was corroborated by the significantly higher number of Osteocalcin-positive cells at 4 weeks in the cell-seeded group. Osteoblasts are specialized cells responsible for bone synthesis and deposition. They are capable of increasing up to 200-fold the amount of Osteocalcin under mineralizing conditions^51^. Osteocalcin is the most abundant non-collagenous protein of the bone extracellular matrix. It is synthesized and secreted only by mature osteoblasts and plays a role in regulating mineralization, and is therefore considered a reliable marker for bone formation and turnover both on clinical and histological settings^51–53^. At 8-weeks, both groups showed a similar TRAP and Osteocalcin expression, which might indicate that the benefit of cell addition is in increasing the early bone remodeling rate, which ultimately results in a higher volume of new bone formation in a shorter amount of time. The definitive origin of osteoclast/osteoblast precursors in the remodeling of grafted material *in vivo* however remains elusive. Our findings suggest that the cells seeded on the microparticulate bone are still viable up to at least 8 weeks and may be playing an active role in bone remodeling. They may possibly differentiate into osteoblast and osteoclast-like cells, while they may also provide chemotaxis for the host cells, and the evaluation of the underlying mechanisms will require further investigations.

This study demonstrated the advantages of using a viable cellularized bone biomaterial in the context of alveolar ridge augmentation compared to acellular bone-derived microparticles. In our animal model, the cell-seeded bone biomaterial showed positive osteogenic properties resulting in significantly more new bone formation together with a higher bone remodeling rate and an overall better incorporation. Future studies will focus in more details on the mechanisms (cell replacement and/or modulation of the host micro-environment) underlying the faster substitution and larger volume of new bone formation we observed and further evaluation in well-characterized larger animal models will be needed to confirm its clinical relevance for craniofacial surgeries and other conditions requiring bone augmentation.

## Materials and Methods

### Animal groups

This study was approved by the University of Miami Institutional Animal Care and Use Committee (IACUC) and all methods were performed in accordance with the relevant guidelines and regulations. Twenty athymic nude rats (NTac:NIH-*Foxn1*^*rnu*^, female, 10 weeks old, weighting 150–200 g) were used in this study. Power analysis and sample size calculations were carried out using G*Power 3.0 software^54^. The power analysis using a One-Way ANOVA experimental design including 4 independent groups with α = 0.05, an effect of 0.85 and a sample size of 20 animals (for all the groups) yields a power of >0.80. The animals were randomly assigned into 2 different groups of 10 individuals with 2 time points each (n = 5 animals per time points). The cell-seeded biomaterial used in the test group (ViaGraft^®^, Vivex Biomedical, Miami, FL) was made of human cadaveric bone microparticles (30% cortical bone, 30% cancellous bone and 40% fully demineralized cortical bone) ranging from 100–300 microns in size and seeded with a DMSO-free cell population that comprises bone marrow-derived MSCs. This product contains on average 220,000 cells/cc of material. The exact same bone microparticles hydrated with DPBS, but with no cells, were used as control (acellular biomaterial group). The macromorphological consistency, texture and appearance of both materials after preparation were indistinguishable. The cells were isolated from cadaveric vertebral bodies bone marrow. Vertebrae cancellous bone was fragmented into small pieces (5–10 mm in diameter) in saline buffer and extensively washed. Cells recovered from the washes were submitted to gradient centrifugation (Ficoll Hypaque) and characterized by flow cytometry (see Supplementary Fig. S1 for CD marker expression) prior to freezing in a DMSO-free cryoprotectant. Cell preparations were used only when the viability was higher than 80% after thawing.

### Rat model of mandibular augmentation: surgical procedure

All surgeries were conducted by the same experienced oral surgeon (Daniel Deluiz) (Fig. 1). The rats were anesthetized with Isoflurane and then placed on a 37 °C heating pad. Puralube^®^ Vet Ointment was applied to the eyes to avoid drying. Antisepsis was made on the submandibular area with topical polyvinylpyrrolidone iodine. The submandibular surgical approach was performed through a linear incision involving the cutaneous and subcutaneous layers exposing the masseter muscle. The muscle was incised along the submandibular border taking care not to injure the facial nerve. A flap including the muscle and periosteum was raised exposing the lateral aspect of the rat’s mandible, thus creating a pouch underneath the masseter. The host bed was kept intact to avoid fracture or insufficient bone to stabilize the titanium screw. One 4.0 mm long, 1.5 mm diameter titanium microscrew (KLS Martin, Tuttlingen, Germany) was fixed on the lateral side of the mandible in order to maintain the space and to stabilize the graft using the tent-pole technique^55^. 0.1cc of the material (cell-seeded or acellular biomaterial) was then placed around and on top of the screw. The amount of material (0.1cc) was chosen to completely fill the void between the bone and the elevated muscle and was then covered with a resorbable amniotic membrane (Cygnus Solo^®^, Vivex Biomedical Inc.). Previous experimental surgeries were performed in animals not included in the study to define the strategy and standardization of the material application. Supplementary Fig. S2 shows a micro-CT scan performed 2 days after surgery to represent the initial graft structure. The placement of such a membrane barrier has been shown by others to be of benefit for new bone formation and was thus used in both groups in our study^48,56–58^. Wound closure was made on the muscle and cutaneous planes with Vicryl 5–0 sutures. Antibiotic therapy was carried out with a single dose of Gentamycin (5.0 mg/kg IM), and buprenorphine (0.1 mg/kg SC) was administered once before surgery and 3 days after for pain control. The rats received a postoperatively soft diet for 3 days and then were returned to the standard food pellets. Water was provided *ad libitum*. The animals were euthanized at 4 and 8 weeks using CO_2_ inhalation and decapitated. Each head/sample was assigned a reference number to blind the examiner to the analyses.

### Micro-CT scan analysis

The animal heads were scanned using a Bruker micro-CT apparatus (SkyScan 1176, Bruker, Kontich, Belgium). The imaging parameters were: 1 mm aluminum filter, exposure of 71 ms, 65 kV, 385 µA, 18 µm pixel size and 0.70° of rotation step. The images were reconstructed using the NRecon software (Bruker) with 30% of beam hardening correction. The analyses were performed by the same investigator presenting an intra-examiner reproducibility Kappa index of 0.90. Animal identification was masked from the investigator. All datasets were aligned to the same orientation (lateral side of the mandible aligned horizontally, and titanium screw aligned vertically) using the DataViewer software (Bruker). For illustrative purposes, an image was reconstructed in a 3D modeling software, VoximOsteo (IVS Technology GmbH, Chemnitz, Germany) (Fig. 2A). A region-of-interest (ROI_1_) comprising the entire grafted area (homogeneous calcified formation around the titanium screw plus spread microparticles resulting from excess material) and excluding the rat’s mandible (and the titanium screw) was selected on the reconstructed images using the CTAnalyser software (Bruker) (red dotted line on Fig. 2B). To avoid the analysis of the excess material which was not participating in bone augmentation, a new region within the ROI_1_ was selected - ROI_2_ (in blue on Fig. 2C). ROI_2_ was defined in a standardized manner for all samples using a threshold of Hounsfield Unit (between +200 and +2000 HU). This threshold included the visually incorporated area (homogeneous calcified formation around the titanium screw) and excluded the excess particles (and the screw) from the measurements. The parameters assessed in the ROI_2_ were: gained bone volume (BV) and bone mineral density (BMD).

### Histology

Immediately after the micro-CT scan analysis, the left mandibles were surgically removed together with the surrounding tissue and fixed with 10% neutral buffered formalin for 1-week. The titanium screws were removed taking care not to damage the grafted areas. The samples were decalcified with Cal-Ex decalcifying solution (Fisher Scientific, Massachusetts, USA) for 3 days, rinsed in distilled water, dehydrated in alcohol, diaphanized in xylene and embedded in paraffin. Paraffin block samples were then sliced at a 3 µm thickness, and then stained with Hematoxylin & Eosin and Masson’s Trichrome for histological and histomorphometrical evaluation. For the Masson’s Trichrome staining, Weigert’s iron hematoxylin, aniline blue and acid stains (fuchsin and xylidin red) were used. The samples were deparaffinized and left in Weigert’s iron hematoxylin for 10 minutes, fuchsin for 4 minutes, phosphomolybdic acid for 5 minutes and in aniline blue for 5 minutes. Washings with distilled water were done before each change of solution. Images were acquired using a Nikon Eclipse 90i light microscope.

### Histomorphometry

The histomorphometric evaluation was performed by a blinded single trained investigator using 4 pictures from each sample (enough to cover the entire grafted area) at 10x magnification using ImageJ (NIH) software. The investigator had been previously tested for intra-examiner reproducibility presenting a Kappa index of 0.80. Newly formed bone as well as unincorporated graft remains were visually identified in the pictures and semi-automatically marked with the software’s selection tool. Errors in the automatic selection were checked and corrected manually. The defined areas of new bone were then colored in blue, while areas of unincorporated biomaterial were colored in red. The color labeling allowed the selections to be more easily distinguishable. The amount of each parameter was calculated as a percentage from the entire image surface area (Fig. 4).

### Immunohistochemical analysis

An anti-OCN (Osteocalcin, osteoblast marker), antibody (clone #: AB10911, Millipore, Massachusetts, USA), diluted in a 1:200 proportion, was used to evaluate new bone formation. An anti-TRAP (Tartrate-resistant Acid Phosphatase, osteoclast marker) antibody (clone #: EPR15556, Abcam, Massachusetts, USA), diluted in a 1:200 proportion, was used to evaluate the remodeling of the grafted biomaterial. An antibody against human mitochondria (hu-Mito clone #: 113-1, Millipore, Massachusetts, USA) was used to identify the cells of human origin in the biomaterial (1:50 dilution). Antigen retrieval was performed in a 10 mM buffered sodium citrate solution. Diaminobenzidine was used as the chromogen and the slides were counterstained with hematoxylin. Using light microscopy, 10 fields of view (FOV) of each slide (covering the entire grafted area) were taken at 40x magnification to quantify the number of TRAP- and OCN-positive cells. The cells were counted within the area of each FOV and the mean count was calculated for the corresponding sample (each sample cell count is a result of the mean of its FOVs). The mean cell count for each group was compared between groups^59^.

### Statistical analysis

Data analysis was performed using the SPSS software (IBM analytics). The normality of the samples was tested using the Shapiro-Wilk test. One-way ANOVA and Kruskal-Wallis tests were used to compare parameters between the groups and *p* < 0.05 was considered statistically significant and depicted with an “*” in the figures.

## Supplementary information

Supplementary Figure S1

Supplementary Figure S2
